# Middle East Respiratory Syndrome Coronavirus (MERS-CoV): State of the Science

**DOI:** 10.3390/microorganisms8070991

**Published:** 2020-07-02

**Authors:** Ahmed Mostafa, Ahmed Kandeil, Mahmoud Shehata, Rabeh El Shesheny, Abdallah M. Samy, Ghazi Kayali, Mohamed A. Ali

**Affiliations:** 1 Center of Scientific Excellence for Influenza Viruses, Environmental Research Division, National Research Centre (NRC), Cairo 12622, Egypt; ahmed_nrc2000@hotmail.com (A.M.); Ahmed.Kandeil@human-link.org (A.K.); Mahmoud.Shehata@human-link.org (M.S.); ra_eny@yahoo.com (R.E.S.); 2 Department of Infectious Diseases, St. Jude Children’s Research Hospital, Memphis, TN 38105, USA; 3 Entomology Department, Faculty of Science, Ain Shams University, Abbassia, Cairo 11566, Egypt; samy@sci.asu.edu.eg; 4 Department of Epidemiology, Human Genetics, and Environmental Sciences, University of Texas, Houston, TX 77030, USA; 5 Human Link, Baabda 1109, Lebanon

**Keywords:** MERS-CoV, epidemiology, coronavirus, zoonotic disease

## Abstract

Coronaviruses belong to a large family of viruses that can cause disease outbreaks ranging from the common cold to acute respiratory syndrome. Since 2003, three zoonotic members of this family evolved to cross species barriers infecting humans and resulting in relatively high case fatality rates (CFR). Compared to Severe Acute Respiratory Syndrome Coronavirus (SARS-CoV, CFR = 10%) and pandemic Severe Acute Respiratory Syndrome Coronavirus 2 (SARS-CoV-2, CFR = 6%), the Middle East Respiratory Syndrome Coronavirus (MERS-CoV) has scored the highest CFR (approximately 35%). In this review, we systematically summarize the current state of scientific knowledge about MERS-CoV, including virology and origin, epidemiology, zoonotic mode of transmission, and potential therapeutic or prophylactic intervention modalities.

## 1. Introduction

Coronaviruses (CoVs) belong to the Coronaviridae family and Nidovirales order. They are enveloped, positive-sense, single-stranded RNA viruses, with the largest genome of all RNA viruses (26–32 kb) [1]. Based on their genetic and antigenic relationships, the International Committee for Taxonomy of Viruses (ICTV) classified CoVs into four genera: Alpha-, Beta-, Gamma-, and Delta-CoVs [2]. CoVs have the capability of interspecies transmission to induce asymptomatic to serious infections in humans, bats, mice, poultry, pets, pigs, and cattle, causing mainly respiratory and enteric diseases [3].

A novel human coronavirus, the Middle East Respiratory Syndrome Coronavirus (MERS-CoV), was first identified in September 2012 in the Kingdom of Saudi Arabia (KSA) in a patient with fever, cough, expectoration, and dyspnea [4]. Since the first case, MERS-CoV has spread globally, causing 2499 human infections including 861 deaths in 27 countries (Asia, Europe, Africa, and North America) as of December 2019 [5,6]. Most cases were reported in countries of the Arabian Peninsula, especially KSA [7,8]. This MERS-CoV outbreak reminded the international public health authorities of the Severe Acute Respiratory Syndrome outbreak of the early 2000s that was caused by another CoV, the Severe Acute Respiratory Syndrome Coronavirus (SARS-CoV) [9]. Even though both SARS- and MERS-CoV belong to the same family, the SARS-CoV outbreak in 2003 was associated with a high incidence rate due to its efficient human-to-human transmission, but low case fatality rate (10%). In contrast, MERS-CoV was relatively inefficiently transmitted between humans but had a high case fatality rate of 35% [10,11].

Two clades of MERS-CoV (i.e., Clade A and B) were recognized based on phylogenetic analysis of viral genomes [12]. The viral genomes detected in the earliest cases in humans (clade A cluster; EMC/2012 and Jordan-N3/2012) were genetically distinct from viruses in clade B circulating in most cases identified later [13]. Recently, an additional clade was identified from the African MERS-CoV strains collected from camels in Africa [14].

Bats have been recognized as a reservoir of most zoonotic coronaviruses [15,16]. MERS-CoV was closely related to bat coronaviruses, supporting the hypothesis that the MERS-CoV infection is zoonotic, where the virus is maintained in an animal reservoir [17,18,19]. Dromedary camels (*Camelus dromedarius*) were also identified as intermediate hosts allowing the MERS-CoV to spread to humans via airborne transmission [20]. High seroprevalence of MERS-CoV was identified in domestic camels [13,14,21,22,23,24,25,26,27,28,29,30,31,32]. The latter evidence was confirmed in camel samples collected from KSA, Qatar, United Arab Emirates (UAE), Oman, Jordan, Egypt, Nigeria, Kenya, Tunisia, Morocco, Burkina Faso, Ethiopia, Israel, and Spain [13,14,21,22,23,24,25,26,27,28,29,30,31,32]. Circulation of MERS-CoV in dromedary camels may drive the transmission in human populations.

Numerous MERS-CoV cases have developed severe symptoms approaching respiratory and kidney failure; however, some MERS-CoV infections appeared asymptomatic [33]. The elevated virulence of MERS-CoV is associated with high case fatality rate (approximately 35%) according to the WHO and FAO official reports [5,6]. Nevertheless, the MERS-CoV mortality rate is overestimated owing to lack of data on asymptomatic and mild cases.

## 2. MERS-CoV Origin of Transmission

The origin and transmission scheme of MERS-CoV remain unknown. The bat origin of MERS-CoV was a strong hypothesis considering the high genome relatedness between sequences of bat coronaviruses and MERS-CoV [34]. Both bat-CoVs (HKU4) and MERS-CoV have the same receptor DPP4 in cell tropism features [35,36,37] Interestingly, MERS-CoV full genome isolation from bats has not been recorded until now, so bat to human transmission of the virus is uncertain.

**Figure 1:**
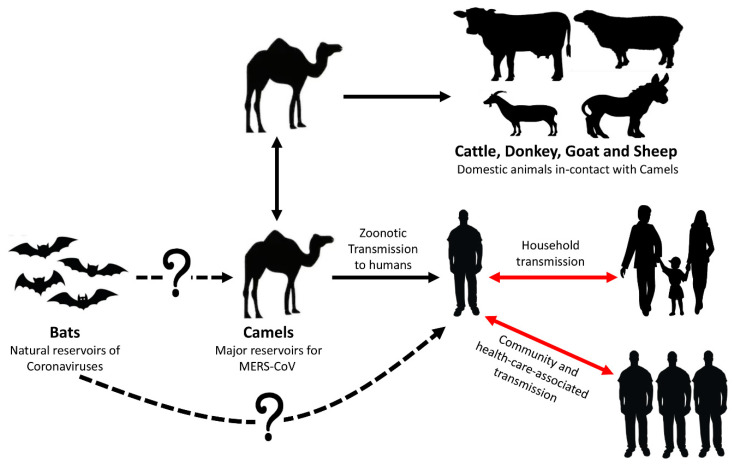
Zoonotic mode of transmission of MERS-CoV. The three routes of transmission (camel-to-camel, camel-to-human, and human-to-human) were confirmed. The prevalence of MERS-CoV in domestic animals in-contact with camels was recently identified [43]. As depicted by the dotted line, bat-to-camel and bat-to-human direct transmission of MERS-CoV have not been confirmed. Human-to-human transmission of the virus occurs after close contact with an infected case in households and healthcare settings (red arrows).

The epidemiological studies in KSA showed that human antibodies against MERS-CoV were still at low prevalence [38,39]. Out of 10,009 individuals of the general population of KSA, only 15 were seropositive (0.15%) [40]. In another report, seven subjects (3.1%) of 87 camel workers and 140 slaughterhouse workers were confirmed positive for MERS-CoV antibodies [41]. Surprisingly, 5000 pilgrims from 22 different nations, who travelled to KSA for Hajj in 2013, were diagnosed negative by PCR for MERS-CoV infection [42]. In the light of current knowledge, the MERS-CoV transmission scheme and origin are illustrated in Figure 1.

Until now, dromedary camels have likely been the main zoonotic source for human infections. MERS-CoV antibodies have already been detected in nearly all dromedary camels examined in the Arabian Peninsula and several African countries [22,23,24,31,44,45,46,47]. Camels in other parts like Europe, Australia, and Americas do not have MERS-CoV antibodies and have no evidence of infection [26,48].

Some human infections with MERS-CoV were linked to exposure to camels; the first evidence was a study in KSA in which the full genome sequences of MERS-CoV were identical in isolates from both a human case with a fatal infection and his camels [49]. These results suggest that MERS-CoV can infect dromedary camels and can be transmitted from infected camels to humans by direct close contact [49]. Furthermore, phylogenetic analyses of the MERS-CoV genome from camel and human isolates demonstrated that the viruses were highly identical or in some cases were similar to each other [13,50,51].

## 3. Epidemiology of MERS-CoV in Animals

### 3.1. MERS-CoV Infection in Dromedary Camels

Mild disease of the upper respiratory tract (URT) or no apparent disease has been observed in young and adult camels naturally infected with MERS-CoV [13,52]. However, experimental infections indicated that MERS-CoV in adult dromedaries results in a mild respiratory disease with purulent nasal discharge [53]. Although clinical signs of the MERS-CoV infection in camels are benign, camels shed large quantities of virus from their URT [52,53]. Experimentally, Adney and his colleagues detected infectious MERS-CoV particles and viral RNA in nasal secretions between 7 days and 35 days post-infection [53]. MERS-CoV genome was detected in nasal swabs of dromedaries in the Arabian Peninsula, Egypt, Iran, Israel, Pakistan, Senegal, and Tunisia [24,54], and the virus was isolated from dromedaries in Egypt, KSA, and Qatar [27,55]. For instance, MERS-CoV RNA has been detected in camel nasal swabs (*n* = 96, positivity = 29.2%) and lung tissue samples (*n* = 91, positivity = 61.5%) during April 2013–May 2014 in Al-Ahsa Province, KSA [56]. This high prevalence of MERS-CoV in camels is attributed to the abundance of the cellular dipeptidyl peptidase 4 (DPP4), which is responsible for binding with MERS-CoV spike protein to initiate the infection, in the URT epithelium of camels [53,55].

### 3.2. Sero-Prevalence of MERS-CoV in Domestic Animals

Serological studies on various animal species in the Middle East were carried out to assess zoonotic potential of MERS-CoV infections [26]. Dromedary camels (*Camelus dromedarius*) appeared to be the source of MERS-CoV. Camel sera from Oman, Canary Islands, and Egypt were positive for MERS-CoV antibodies in about 100%, 14%, and 90%, respectively [29,30,46]. Retrospective studies on archived human sera showed no evidence for infection with MERS-CoV before 2012 [39], but MERS-CoV antibodies were detected in archived camel sera in KSA from 1993 [26], and UAE from 2003 [25], indicating that MERS-CoV viruses were silently circulating in camels long before they were discovered in 2012. Bactrian camels in Mongolia tested negative for MERS-CoV antibodies [57]. The serological studies in domestic animals in contact with camels in the same housing area showed antibodies against MERS-CoV in Egypt, Tunisia, and Senegal [43,58]. The serological evidence of MERS-CoV prevalence in dromedary and domestic animals are listed in Table 1.

## 4. Epidemiology of MERS-CoV in Humans

Unlike camels, the human URT lacks DPP4 expression, and this may be the primary cause of limited MERS-CoV replication and hence restricted human-to-human transmission [53]. Most human infections have been reported in KSA (2073 cases; case fatality rate = 37%) and UAE (87 cases) with an evidence of case clusters in these countries [6,70,71,72]. Cases outside the Arabian Peninsula have been documented in Europe, Asia, North Africa, and North America, and were reported in travelers returning to their countries from the Arabian Peninsula region [73,74,75].

### 4.1. Distribution Pattern and Human Infections in Middle East

In September 2012, the MERS-CoV was first isolated from a Saudi citizen (60 years old), who manifested kidney failure and acute pneumonia. Afterwards, a case from Qatar was reported with the same symptoms and who had a travel history to KSA [4,76,77]. Many cases have been subsequently reported from Middle East countries [78].

MERS-CoV has been largely detected in the Middle East region, especially in the Arabian Peninsula, where large numbers of dromedary camels are reared. The serological and virological inspections of camels in Iran, Iraq, Israel, Jordan, Kuwait, Oman, Qatar, KSA, and UAE [79] revealed the highest distribution of MERS-CoV in this area. Reeves and colleagues revealed a preliminary map of MERS-CoV; climatic dimensions of camel-associated cases are more constrained and less variable than the broader suite of MERS-CoV occurrences [80]. The latter findings reflected the importance of camel exposure as a key limiting factor for virus circulation.

### 4.2. Distribution Pattern and Human Infections in Africa

MERS-CoV antibodies have been reported in camels in various African countries including Burkina Faso, Egypt, Ethiopia, Kenya, Mali, Morocco, Nigeria, Senegal, Somalia, Sudan, Tunisia, and Uganda [54,66]. This emphasizes that MERS-CoV circulation among dromedaries in different geographical localities of Africa is extensive, and the viral shedding is as common as frequently reported in the Arabian Peninsula [54]. Despite this high prevalence of MERS-CoV in camels in Africa, the actual number of cases was relatively low including in Tunisia (3 cases), Algeria (2 cases), and Egypt (one case); all were travel related to the Arabian peninsula [78]. No index human cases were detected in Africa.

### 4.3. Viral Invasion to Asia, Europe, and America

Seropositive animals for MERS-CoV were reported in Asia (e.g., Bangladesh and Pakistan), Europe (e.g., Canary Islands, Spain), and Africa [45,59,60,68,69]. Nevertheless, all human cases reported in China, Thailand, Malaysia, Philippines, United Kingdom, Austria, Greece, the Netherlands, France, Germany, Italy, and the United States were imported via travelling from Middle East countries [5,6].

In May 2015, an outbreak of MERS-CoV occurred in South Korea, resulting in a total of 186 human cases and a 19.35% mortality rate. The average age of Korean cases recorded were 55 years (ranging from 16 to 87 years), and 14% of cases in the South Korean outbreak were healthcare workers. The first case was an elderly male (68 years) who reported multiple travels to several countries of the Arabian Peninsula [74]. Later, MERS-CoVs were also reported in human cases from the Philippines (2 cases), Thailand (3 cases), China (one case), and Malaysia (2 cases) [78].

### 4.4. Clusters of MERS-CoV Infections

Despite the fact that the MERS-CoV was firstly reported in KSA in September 2012, CDC identified retrospectively MERS-CoV as the etiological agent responsible for two fatal cases of respiratory illness in Jordan in April 2012. Therefore, April 2012 is likely marked as the onset of the first MERS-CoV outbreak in humans [81,82]. Concurrently, in September 2012, another man from Qatar with a severe respiratory illness was admitted to a hospital unit in the United Kingdom. His case history revealed that he firstly developed illness in Qatar, traveled to KSA, and then to the UK for remediation [77,83,84].

The first cluster of MERS-CoV infections was in November 2012 when a family contracted the virus in Riyadh, KSA. A total of 24 family members who had been in contact with the index case or with healthcare workers developed the illness, and two of the sick died [35].

The second cluster was retroactively reported in Jordan in 2012 and was related to the earliest confirmed two fatal cases (seven subjects including six healthcare workers) [81].

From 1st of April to 23rd of May 23, 2013, a total of 23 cases of MERS-CoV infections were reported in Al-Hasa governorate in the eastern province of KSA. Interestingly, a total of 21 of the 23 cases were acquired via person-to-person transmission in hemodialysis, in-patient, or intensive care units of three different healthcare facilities. Additionally, five laboratory-confirmed MERS-CoV cases were documented among 217 household and more than 200 healthcare workers [85] in the three different healthcare facilities, confirming that the person-to-person transmission of MERS-CoV can occur in healthcare settings and may be associated with considerable morbidity [85].

A patient infected with MERS-CoV with travel history to UAE was reported in France in May 2013. This patient shared a room in the hospital with another patient that became infected. The index case died and the second patient recovered [86].

MERS-CoV infections rapidly increased in KSA and the UAE during March and April 2014 [72,87]. Most of the cases were identified from outbreaks in hospitals in Riyadh, Jeddah, Madinah, and Tabuk in KSA, and in Al-Ain and Abu-Dhabi in the UAE. Cases included hospital workers, patients, visitors, and ambulance member staff. Asymptomatic or mild symptomatic infections were noticed in the majority of hospital workers but only 15% ended up with severe sickness or death [72]. The first case in the USA was reported in an American healthcare worker working in Riyadh, KSA. He went to Indiana in April 2014, and was admitted to the intensive care unit in the hospital [88]. The second reported case in the USA was diagnosed positive for MERS-CoV in May 2014 in Florida in an American citizen who had shortly returned from KSA [88,89].

The largest outbreak in South Korea started in May 2015 [75]. The first case was reported in a man who was on a journey in Bahrain, UAE, KSA, and Qatar [72,74]. Since May 2015, more than 180 associated cases were reported, including 36 deaths from both family members and hospital visitors [75]. Meanwhile, the first MERS-CoV case reported in China was in a Chinese man who traveled from South Korea to China [72,74].

## 5. Impact of Comorbidities on MERS-CoV Infections in Humans

Comorbidities such as asthma, diabetes mellitus, hypertension, ischemic heart disease, congestive heart failure, end-stage renal disease, and chronic kidney disease are known to weaken the host’s innate and humoral immune systems and impair the production of proinflammatory cytokines, thereby limiting their ability to counteract any new infection [90]. Consequently, most fatalities have been documented in hospitalized patients with pre-existing comorbidities [91]. A systematic analysis of 637 MERS-CoV cases deduced that diabetes and hypertension are equally prevalent in approximately 50% of the patients. Cardiac diseases are present in 30% and obesity in 16% of the cases [92]. Statistically, the existence of underlying comorbidities was found to significantly complicate the infection with MERS-CoV, influence its severity, and significantly increase the overall fatality rates [93]. MERS patients of comparable age with comorbidities had approximately four times the risk for fatal infection as those without any comorbidity within the same epidemic period [94]. Additionally, the MERS-CoV DPP4 receptor is upregulated in the lungs of cigarette smokers and patients with chronic obstructive pulmonary disease (COPD), and this upregulation could explain why smokers and patients with comorbid lung diseases are more susceptible to MERS-CoV infection and are subject to severe MERS disease [95].

## 6. Evolution of MERS-CoV

Human CoVs (HCoVs) are of the alpha and beta genera of CoVs. MERS-CoV belongs to clade C of the beta-CoV genus [2,36]. Beta-CoVs, *Tylonycteris* bat virus (HKU4) and *Pipistrellus* bat virus (HKU5) are suggested as the closely-related species to MERS-CoV in clade C [96]. Additionally, a *Neoromicia zuluensis* bat virus was another related MERS-CoV in South Africa [97]. This highlighted the hypothesis that *Pipistrellus* and *Neoromicia* genera in the Vespertilionidae family were the reservoirs of MERS-CoV ancestors [34]. A rooted phylogenetic tree of MERS-CoV indicates that MERS-CoV first emerged in camels before zoonotic transmission to humans [34].

**Figure 2:**
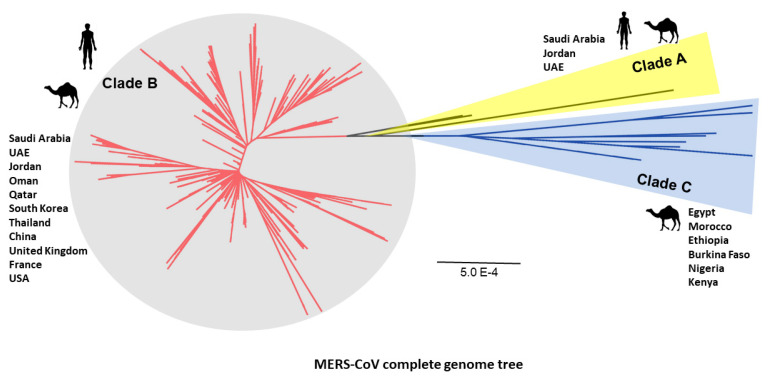
Three clades of MERS-CoV based on a rooted phylogenetic tree of 484 complete genomes of MERS-CoV strains from camel and human cases. MERS-CoV isolates are divided into three separate clades: A, B, and C. Clades A and B are prevalent in the Arabian Peninsula and other non-African world countries. Clade C is mainly circulating in African countries. The optimal tree with the sum of branch length = 0.11869958 is shown with scale bar = 0.0005 (5.0E−4).

In this review, all available complete genomes were collected from the MERS-CoV database for human and camel isolates [98]. A rooted phylogenetic tree showed diverse MERS-CoV clades (Figure 2). MERS-CoV isolates were phylogenetically distinguished into three separate clades: A, B, and C. Clade A comprises the first EMC/human strain in KSA, Jordan-N3/2012 of 2012 and UAE camel strain [4,99,100]. MERS-CoV camel strains from Egypt, Morocco, Ethiopia, Burkina Faso, Nigeria, and Kenya were found in clade C [14,55,101]. The rest of human and camel strains mainly in the Arabian Peninsula and other countries with travel related to Arabia were sorted into clade B (Figure 2).

## 7. Mutation Patterns in Spike Protein of MERS-CoV

**Figure 3:**
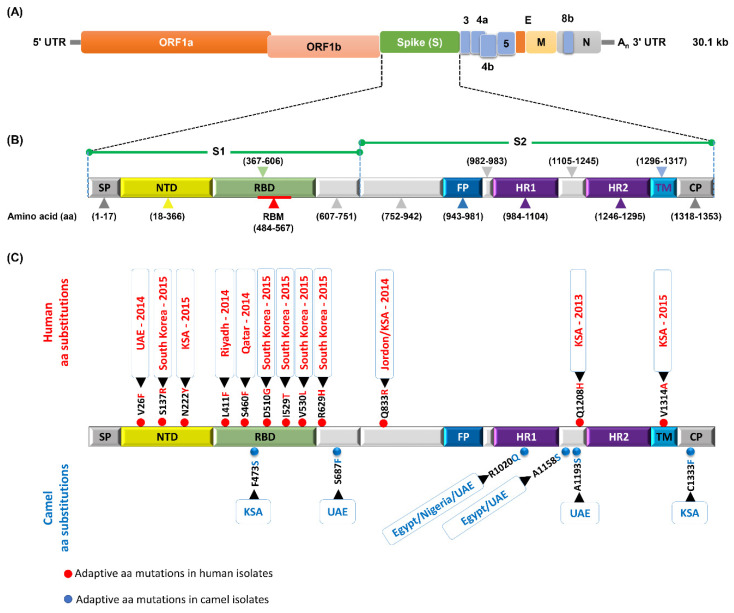
Schematic diagram of the MERS-CoV genome and naturally selected aa substitutions in spike protein. (**A**) The genomic structure of MERS-CoV (30.1 kb in length), illustrating sub-genomic viral RNA transcripts. (**B**) Schematic structure of the MERS-CoV S protein and its functional domains, including the N-terminal domain (NTD), receptor-binding domain (RBD), receptor-binding motif (RBM), fusion peptide (FP), heptad repeat region 1 and 2 (HR1 and HR2), transmembrane region (TM), and cytoplasmic tail (CP). (**C**) Since the first documentation of MERS-CoV in 2012 in KSA, the virus circulated in camels and occasionally humans to naturally acquire distinct adaptive amino acid (aa) substitutions.

The MERS-CoV genome is approximately 30.1 kb in size and generally encodes (1) structural spike (S), nucleocapsid (N), membrane (M), and envelope (E) proteins; and (2) nonstructural accessory (replicase (ORF1a and ORF1b), ORF 3, ORF 4a, ORF 4b, ORF 5) proteins (Figure 3a). The S protein is a glycosylated type I membrane protein that decorates the crown shape of the virion and functionally recognizes the cellular protein DPP4 via its receptor binding domain (RBD) to initiate viral entry into target cells.

**Figure 4:**
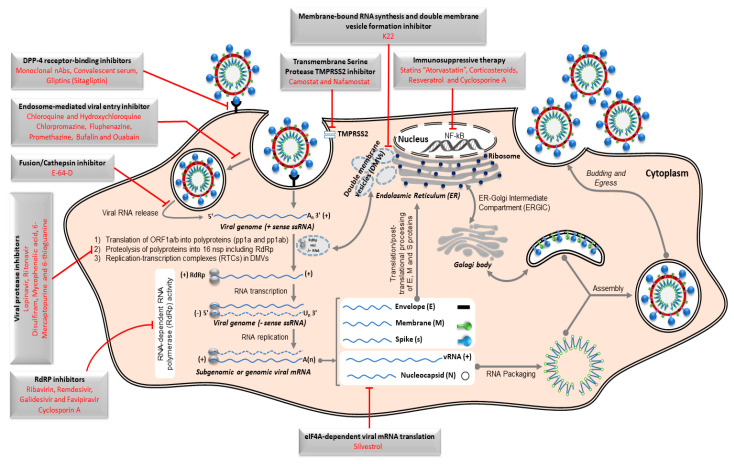
Schematic representation of Middle East respiratory syndrome coronavirus (MERS-CoV) replication cycle and key targets for antiviral activity. The spike protein of MERS-CoV initiates host cell infection via binding its receptor-binding domain (RBD) in the S1 subunit into cellular receptor dipeptidyl peptidase 4 (DPP4), originally known as the lymphocyte cell surface protein CD26. Following binding, the viral particle in the form of an endosome internalizes into the cytosol via acid-dependent proteolytic cleavage of S protein by a cathepsin or TMPRRS2. To release the viral genome (+ssRNA) into the cytoplasm, fusion of the viral and cellular membranes within acidified endosome occurs. Initially, the replicase gene, which encodes the largest two open-reading frames “ORF1a and ORF1ab”, is translated to express two co-terminal polyproteins, pp1a and pp1ab. These polyproteins are further cleaved by virus-encoded proteases “papain-like protease PLpro and 3C-like protease 3CLpro" into 16 mature nonstructural proteins (nsp) including viral polymerase subunits. Essential elements for viral genome replication are gathered as RNA replication–transcription complexes (RTCs) within the endoplasmic reticulum-derived double-membrane vesicles (DMVs). The RTCs drive the production of intermediate negative-sense viral genome (–ssRNA) transcript. During replication, the –ssRNA genome is used as a template for generating nascent +ssRNA. Along with the continuous transcription to generate the nascent full-length coding +ssRNA, sub-genomic RNAs, including those encoding all essential structural proteins (spike (S), envelope (E), membrane (M), and nucleocapsid (N)), are produced via discontinuous transcription. Nucleocapsid and nascent genomic RNA are assembled together in the cytoplasm and further transported into the lumen of the endoplasmic reticulum (ER)–Golgi intermediate compartment (ERGIC). Meanwhile, the S, E, and M sub-genomic RNAs are translated and inserted into the membrane of the rough endoplasmic reticulum (ER), from where they are transported to interact with RNA-encapsidated N proteins in the ERGIC, forming a mature viral particle. Via exocytosis, the nascent viral particle is then released from the infected cell. The repurposed therapeutic drugs undergoing preclinical and clinical trials against MERS-CoV in the context of host pathways and virus replication mechanisms are represented in the figure. The symbol ⊣ refers to inhibition.

The functional domain of MERS-CoV S protein comprises the N-terminal domain (NTD), receptor-binding domain (RBD), receptor-binding motif (RBM), fusion peptide (FP), heptad repeat region 1 and 2 (HR1 and HR2, respectively), transmembrane region (TM), and cytoplasmic tail (CP) (Figure 4b). The genetic alterations in the spike protein, especially in the RBD, may alter the virus transmissibility from one host to another. Consequently, following up the genetic and antigenic variations in the MERS-CoV spike protein is pivotal to recognize the molecular determinants of virus evolution and transmissibility. Moreover, recent studies have shown that several amino acid (aa) mutations were probably responsible for immune evasion of MERS-CoV [102]. During the outbreak in South Korea, the aa substitutions D510G and I529T in the RBD region were observed in most human MERS-CoV strains [103]. Evolution and transmission of MERS-CoV from bats to humans and camels may be acquired due to six mutations in the RBD region at sites Q419S, G436N, G472S, R479L, K511R, and G521N [104]. A lot of mutations in the spike protein were associated with major MERS-CoV outbreaks in KSA, UAE, and South Korea (Figure 3).

For instance, the L411F substitution in the RBD region was found in strains from an outbreak in Riyadh, KSA in 2014 and in camel strains in Jeddah, KSA region [105]. S460F is unique in two human strains from Qatar. F473S is exclusively found in camel strains in Jeddah and Riyadh [105,106]. In the fusion peptide region, Q833R appeared in human Jeddah strains in 2014. V810I was prevalent in camel strains in the UAE, KSA, and Oman human cases [28]. In the N-terminal domain (NTD), V26A is a unique mutation found in camel strains of Egypt, Nigeria, and UAE in clade C, and V26F was found in human strains from Abu Dhabi, UAE in 2014 in clade B. H194Y was remarked in all clade A strains except in the EMC-human strain of 2012. N222Y was found in Buraydah, KSA human infections in 2015 in clade B. The contribution of these aa substitutions to alterations in receptor binding affinity, replication efficiency, and pathogenic significance has not yet been precisely evaluated and demands further effort to be investigated.

In the heptad repeat 1 (HR1) region, R1020Q marked camel strains in Egypt, Nigeria, UAE, and some KSA human strains in Jeddah [107,108]. A1193S in the region between HR1 and HR2 in the S2 fragment was predominant in clade B viruses that caused infections among healthcare workers in Abu Dhabi in 2014 [109]. A1158S was detected in camel strains in Egypt, UAE, and two human Jordanian cases. Q1208H also appeared as a unique mutation related to the Al-Hasa, KSA human outbreak in 2013 [12]. In the trans-membrane domain (TM) region, V1314A was observed in 2015 human cases in Riyadh, KSA in clade B. Additionally, C1333F in the cytoplasmic domain (CP) was observed in Jeddah camel strains through 2014 and 2015.

Other aa substitutions were documented to emerge during tissue-culture or mouse adaptation of MERS-CoV, and resulted in an improved replication efficiency in vitro and higher pathogenicity in vivo [110,111]. S746R and N762A are critical for bat-to-human transmission via mediated viral entry into human cells [110].

## 8. Control Approaches for MERS-CoV

### 8.1. Antiviral Remedy

Currently, no effective therapeutic interventions are approved or certified for treating the severe and acute infections with MERS-CoV or other coronaviruses. To rapidly identify potential therapeutic options against MERS-CoV, testing existing FDA licensed drugs for efficacy against novel viral pathogens represents a practical approach for antiviral screening (Figure 4). This could expedite the recommendation and/or implementation of those FDA approved drugs with effective anti-MERS-CoV activity in treatment protocols.

To date, numerous pharmaceutically active ingredients have shown potential for suppressing the replication of MERS-CoV in permissive Huh-7 and Vero-E6 cell lines. Those are currently applied as anti-diabetes mellitus type 2 inhibitors of DPP4 (such as sitagliptin), antibacterial agents (emetine dihydrochloride hydrate), antimalarial agents (chloroquine, hydroxychloroquine, amodiaquine, and mefloquine), antiparasitic agents (niclosamide and amodiaquine dihydrochloride dihydrate), antidiarrheal agent (loperamide), sterol metabolism inhibitor (triparanol), anti-cancer drugs (bufalin, imatinib, gemcitabine, and trametinib), protein-processing inhibitors (lopinavir, ritonavir, and disulfiram), neurotransmitter inhibitors (benztropine mesylate, fluspirilene, thiothixene, fluphenazine hydrochloride, promethazine hydrochloride, astemizole, chlorphenoxamine hydrochloride, chlorpromazine hydrochloride, thiethylperazine maleate, triflupromazine hydrochloride, and clomipramine hydrochloride), estrogen receptor antagonists (tamoxifen and toremifene citrate), kinase signaling inhibitors (imatinib mesylate, rapamycin, saracatinib, and dasatinib), and viral RNA-dependent RNA polymerase (RdRp) inhibitors (ribavirin, remdesivir, galidesivir, and favipiravir) [112,113,114,115,116,117,118,119,120,121,122,123].

In addition, severe MERS-CoV infections are likely associated with hyper-immune responses represented by the excessive production of inflammatory cytokines, chemokines, and interferon stimulated genes, namely cytokine storm (CS) [124]. To this point, immunosuppressants and inflammatory cytokines antagonists such as statins, (e.g., atorvastatin), resveratrol, and cyclosporine A are useful to minimize the consequences of CS, such as acute respiratory distress syndrome (ARDS) [121,125].

These several pharmaceutical classes of drugs were proven either in vitro or in vivo to be beneficial to control MERS-CoV infection. Most of these drugs appear to target host factors rather than viral proteins specifically [115]. Further studies should be applied to identify the non-viral targets of these drugs, providing a basis to discover new candidates for future research studies and clinical intervention protocols especially for newly emerging coronavirus infections such as pandemic SARS-CoV-2.

### 8.2. Vaccination

#### 8.2.1. Inactivated Vaccine

The inactivated vaccine development process includes the propagation of the virus vaccine strain to appropriate titers followed by chemical or physical inactivation. This inactivated vaccine is widely applied and commercially traded to protect humans and domestic animals against many pathogens. It has several advantages including low production cost, high safety and stability, lack of post-formulation genetic modifications of candidate vaccine seed strains, and the enrichment of the inactivated vaccine with all viral cross-reactive antigens, which may contribute to improved viral immunogenicity. Contrarily, inactivated vaccine requires high biosafety containment level for virus propagation and antigen preparation, as well as suitable adjuvants to enhance immunogenicity [126]. Formaldehyde-inactivated whole MERS-CoV adjuvanted with Imject Alum induced specific neutralizing antibodies in vaccinated mice after two weeks post-vaccination that dramatically increased by eight weeks post-immunization, providing full neutralizing capacity [127]. Deng et al. showed that vaccinated mice with inactivated MERS-CoV with a combined adjuvant alum with CpG dinucleotides as a DNA vaccine adjuvant elicited a protective immune response during challenge infection in hDPP4 transgenic mice [128]. Moreover, an inactivated chimeric rabies virus displaying glycoprotein (G) with spike protein subunit 1 (S1) protein of MERS-CoV induced robust neutralizing antibody responses against rabies and MERS-CoV [129]. Gamma irradiation (γ)-inactivated MERS-CoV vaccine evoked specific neutralizing antibodies and reduced viral titers in hDPP4 transgenic mice during challenge infection [130]. Mice vaccinated with γ-inactivated MERS-CoV vaccine developed lung mononuclear infiltrates with increased eosinophils promoting IL-5 and IL-13 cytokines, emphasizing that inactivated MERS-CoV vaccine can be accompanied with a hypersensitive-type lung pathology risk following challenge infection [131]. Recently, Shehata et al. generated a recombinant influenza A/H1N1 expressing chimeric neuraminidase protein with a short immunogenic peptide of MERS-CoV. This bivalent vaccine formulation induced potent and specific neutralizing antibodies against MERS-CoV in balb/c mice [132].

#### 8.2.2. Live-Attenuated Vaccine

Live-attenuated vaccines are the most immunogenic and effective vaccines, even without supplementary adjuvant. Several methods were developed to generate candidate live-attenuated vaccine strains including genetically engineered low pathogenic vaccine strains using reverse genetic technology or virus attenuation by successive passaging of the candidate vaccine strain. However, live-attenuated vaccine has several limitations, including (1) the risk of reversion of the avirulent live-attenuated virus to its virulent phenotype during viral replication in the vaccinated host, (2) a necessity for a vaccine cold chain to maintain the vaccine potency, and (3) the inappropriateness of the vaccine for infants, immunocompromised individuals, and elderly people.

The first approach to develop a live-attenuated vaccine against MERS-CoV was achieved by deleting the structural E gene to produce a non-infectious recombinant MERS-CoV “rMERS-CoV ∆E” virus. The developed rMERS-CoV ∆E virus could only be grown in the laboratory by providing E protein in tran [133]. The rescued rMERS-CoV ∆E virus, using reverse genetic technology based on bacterial artificial chromosome (BAC), allowed the generation of the first modified live vaccine candidate to protect against MERS-CoV [133]. In another study, a recombinant measles virus (MV) was used to express the full-length spike protein of MERS-CoV (MVvac2-CoV-S) [134]. The authors showed that the integration of the MERS-CoV spike protein into recombinant MV is genetically stable and induces strong neutralizing antibodies and cellular immunity against MERS-CoV in vaccinated hDPP4-transduced-mice [134].

Another approach to attenuate MERS-CoV was achieved through mutation of the highly conserved nonstructural protein 16 (NSP16) to lose its activity using reverse genetics [135]. The NSP16-deficient (dNSP16) MERS-CoV mutant became sensitive to type I interferon (IFNα/β), providing a clear attenuation mechanism of MERS CoV. The dNSP16 MERS-CoV mutant induced high levels of neutralizing antibodies and provided full protection against lethal MERS-CoV challenge in mice [135]. Live-attenuated bivalent recombinant MV expressing MERS-CoV N protein (MVvac2-MERS-N) induced robust and multifunctional T cell responses against MV and MERS CoV in an appropriate mouse model [136]. Comparing MVvac2-MERS-N and MVvac2-MERS-S vaccines indicated that MVvac2-CoV-S has a higher quality of cellular immune responses [136]. More recently, a live recombinant vesicular stomatitis virus (VSV) displaying the S protein of MERS-CoV instead of the G protein induced both humoral and cell-mediated immunities in vaccinated rhesus macaques after a single dose of immunization [137]. More recently, Kato et al. developed a novel bivalent RVΔP-MERS/S1 vaccine against rabies virus (RV) and MERS-CoV using a replication-incompetent P-gene-deficient RV (RVΔP) [138]. Using a reverse genetics method, the recombinant RVΔP was used to express S1 fused to transmembrane and cytoplasmic domains together with 14 amino acids from the ectodomain of the RV-glycoprotein (RV-G). In RVΔP-MERS/S1- and RVΔP-vaccinated mice, there were no rabies-associated signs or symptoms. Serologically, RVΔP-MERS/S1 induced specific neutralizing antibodies against both MERS-CoV and RV following its intraperitoneal injection [138].

#### 8.2.3. Subunit Vaccine

Among different types of vaccines, subunit vaccines have the highest safety profile in spite of their low immunogenicity [139]. Until now, the approaches to develop a subunit MERS-CoV vaccine were based on the adjuvanted full-length S, S1, receptor-binding domain (RBD), or N-terminal domain (rNTD) of the S protein. The RBD protein of MERS-CoV S is frequently recommended as a candidate antigen to develop MERS-CoV subunit vaccines. An adjuvanted “Montanide ISA 51” recombinant peptide comprising a 212-amino acid fragment (residues 377–588) of RBD (residues 367–606) of MERS-CoV spike protein fused with human IgG Fc fragment (S377-588-Fc) were able to induce strong MERS-CoV S-specific antibodies in vaccinated mice, enabling the binding of the RBD to DPP4 receptor and combating MERS-CoV infection via neutralization [140]. Among five different versions of RBD fragments fused with human IgG Fc, the form S377–588-Fc showed the highest DPP4-binding affinity and induced the highest titer of specific neutralizing IgG antibodies against MERS-CoV in vaccinated mice and New Zealand white rabbits [141]. Interestingly, an adjuvanted S377–588-Fc subunit vaccine elicited systemic humoral immune responses in mice vaccinated via intranasal and subcutaneous routes. Of note, stronger immune responses were observed in immunized mice via intranasal route than those injected subcutaneously [141]. The immunization of rabbits with the S358–588 S1-Fc fragment of RBD evoked specific neutralizing polyclonal antibodies against MERS-CoV [142]. Moreover, recombinant RBD fragment (S377–662) of S1 against (HCoV-EMC/2012) was shown to elicit high neutralizing antibodies in vaccinated mice [143]. In another study, the recombinant RBD fragment, S367–606, provided specific humoral and cellular immunity in BALB/c mice after vaccination [144]. Zhang et al. elucidated the effects of different adjuvants including Freund’s adjuvant, alum, monophosphoryl lipid A, Montanide ISA51, and MF59 on the induction of host immune responses to a MERS-CoV RBD-based subunit vaccine, S377–588-Fc protein, as a model antigen. They demonstrated that the S377–588-Fc protein induced highly potent specific immune responses when formulated with MF59 adjuvant [145]. Mixing of multiple adjuvants together with the S377–588-Fc protein could synergistically improve the efficacy of the RBD-based subunit vaccine [146]. In line with this, Tai et al. showed that the recombinant trimeric RBD protein generated by fusing the RBD sequence (residues 377–588) with Fd trimerization motif induced protective neutralizing antibodies and protected hDPP4 transgenic mice from lethal MERS-CoV challenge [147]. Similarly, Pallesen et al. synthesized a recombinant pre-fusion trimeric MERS-CoV S protein to induce high titers of specific neutralizing antibodies in vaccinated BALB/c mice [148]. A robust neutralizing antibody response was elicited in BALB/c mice against MERS-CoV after immunization with purified full spike (S) protein nanoparticles (approximately 25 nm) produced in SF9 cells infected with specific recombinant baculovirus expressing S protein [149]. In another trial by Jiaming et al., a recombinant rNTD was used as a potential subunit vaccine candidate to induce neutralizing antibodies, and it could reduce the respiratory tract pathology of immunized BALB/c mice in a non-lethal MERS-CoV challenge [150]. Another study showed that adjuvanted (MF59) MERS-CoV S1 protein protected vaccinated mice against lethal infection of MERS-CoV during challenge experiment, where the protection correlated well with the neutralizing antibody titer [151]. More recently, Adney et al. showed that vaccination with an adjuvanted MERS-CoV spike protein as a subunit vaccine provided complete protection against MERS-CoV infection in alpacas and resulted in reduced and delayed viral shedding in the upper airways of infected dromedary camels [152].

#### 8.2.4. Virus-Like Particle (VLP) Vaccine

In general, the VLP vaccine is similar to the inactivated vaccine, but does not require the viral inactivation step which may alter the antigenicity and immunogenicity of a viral protein. Additionally, VLPs can be easily generated in a low-containment manufacturing environment due to the processing of pathogen-free materials [126]. A novel chimeric VLP vaccine displaying a fused canine parvovirus (CPV) VP2 structural protein with RBD of MERS-CoV induced specific humoral and cellular immune responses against MERS-CoV in vaccinated mice [153]. Additionally, Wang and his colleagues generated MERS-CoV VLPs by co-expressing structural S, E, and M proteins of MERS-CoV using the baculovirus expression system [154]. Electron microscopy demonstrated that the developed MERS-CoV VLPs were structurally similar to the native MERS-CoV virus [154]. Alum adjuvanted MERS-CoV VLPs induced high specific IgG antibody titers against the RBD and elicited cellular immunity in intramuscularly vaccinated rhesus macaques [154]. By using a baculovirus insect cell expression system to generate chimeric VLPs, S protein of MERS-CoV and matrix protein 1 (M1) of influenza A virus (IAV) were developed, showing high immunogenicity in mice when adjuvanted with alum and CpG ODN. The adjuvanted chimeric VLP vaccine elicited neutralizing antibody responses, while the actual protective efficacy of this chimeric VLP vaccine against MERS-CoV was not investigated in vivo [146].

#### 8.2.5. Viral Vector-Recombinant Vaccines

Adenovirus, Newcastle disease virus (NDV), vesicular stomatitis virus (VSV), rabies virus (RABV), and modified vaccinia virus Ankara (MVA) were used as successful viral vectors in the development of MERS-CoV vaccines. Unlike inactivated and subunit vaccines, viral vector-recombinant vaccines do not require adjuvants to be immunogenic. Two recombinant human adenoviral vectors (rAd5 and rAd41) encoding the full-length S protein showed neutralizing antibodies in immunized BALB/c mice [155]. The same study examined the effects of intramuscular or intragastric immunization route on immune responses using a single dose of rAd5 or rAd41 vectors encoding S of MERS CoV. Intragastric administration of any recombinant adenoviral vaccine induced humoral immunity; however, cellular immune responses were not detected. In contrast, intramuscular administration of Ad5-S or Ad41-S induced both types of immunity [155]. Recombinant human adenoviral vector 5 (rAd5) encoding full S protein or shorter extracellular S1 domain of HCoV-EMC/2012 isolate induced antibody responses against MERS-CoV in vaccinated BALB/c mice [156]. A recent (2019) study by Hashem et al. revealed that the incorporation of CD40L into rAd5-based MERS-CoV S1 vaccine provided a complete protection to hDPP4 transgenic mice and prevented pulmonary perivascular hemorrhage post-viral challenge [157]. Chimpanzee adenovirus (ChAdOx1) has been used in the development of a viral vector vaccine against MERS-CoV. The recombinant (ChAdOx1 MERS) encoding full-length S protein of MERS CoV induced specific antibody response and offered complete protection to hDPP4 transgenic mice post-MERS-CoV viral challenge by only a single dose of intranasal or intramuscular immunization [158,159]. The recombinant (ChAdOx1 MERS) encoding full-length S vaccine was improved by insertion of a gene encoding the signal peptide of human tissue plasminogen activator (tPA) upstream of the S gene of MERS-CoV [158]. Chimpanzee adenovirus (ChAdOx1) containing the MERS-CoV S glycoprotein antigen has entered human clinical trials and is known as MERS001. This vaccine requires a one-time shot of 5 × 109–5 × 1010 virus particles via the intramuscular route. The phase I clinical trial is sponsored by the University of Oxford, United Kingdom and is estimated to be completed by July 2021 (https://www.clinicaltrials.gov/ct2/show/record/NCT03399578).

Another recombinant viral vector for the development of a MERS CoV vaccine, vaccinia virus Ankara (MVA) encoding full length S protein, showed a good safety profile, immunogenicity, and high protective efficacy [158,160]. Volz and his colleagues developed a recombinant modified vaccinia virus expressing full S protein and immunized Ad5-hDPP4-transduced BALB/c mice. Results showed induction of humoral and cell-mediated immunity and neutralizing antibodies [161]. Another candidate vaccine currently in a phase I clinical trial is MVA-MERS-S (https://clinicaltrials.gov/ct2/show/NCT03615911). The trial is being performed by the University Medical Center Hamburg-Eppendorf, Germany, in which the safety and immunogenicity of MVA-MERS-S in healthy adult volunteers are being assessed. Another study on Ad5-hDPP4-transduced BALB/c mice immunized with Venezuelan equine encephalitis virus replicon particles containing S protein elucidated a reduction of viral titers to nearly undetectable levels after one day post infection and increased neutralizing antibody titers [162]. The vesicular stomatitis virus (VSV) used in a recent study as a viral vector for the spike gene of MERS-CoV formulating chimeric virus called (VSVΔG-MERS) induced immunological T-cell response and virus neutralization in a rhesus monkey model against VSV and MERS-CoV with a single immunization dose [137]. The NDV displaying MERS-CoV S protein vaccine induced neutralizing antibodies in BALB/c mice and Bactrian camels [163].

#### 8.2.6. DNA Vaccine

DNA encoding the full-length S protein was shown to induce neutralizing antibodies and robust cell-mediated immunity in immunized mice, macaques, and camels [164]. Wang et al. developed two candidate vaccines, namely a subunit (full S and S1 protein fraction) and a DNA vaccine (full S and S1 gene in a mammalian VRC8400 vector), which were used to immunize female BALB/c mice and Indian rhesus macaques [151]. Results showed that of the eight vaccine regimens, the full S DNA (plus electroporation) and S1 protein vaccines elicited high titers of neutralizing antibodies in mice against different strains of MERS-CoV. Three vaccine regimens that were more immunogenic in mice were selected to be evaluated in the rhesus macaque model. The full S DNA and S1 protein yielded potent nAb titers in sera of rhesus macaques. Additionally, the DNA-primed regimen induced earlier protection from pneumonia and clearance of lung infiltrates in rhesus macaques due to induction of effector CD8 + T cells [151]. Other researchers showed that immunization of mice with a DNA vaccine encoding the S1 domain and passive immune sera protected hDPP4-transgenic-mice from MERS-CoV infection [165]. The DNA vaccine having partial S showed more immunogenicity than full-length S; however, both DNAs encoding the S1 and S proteins were shown to induce neutralizing antibodies that cross-reacted with MERS-CoV strains of human and camel origins [166].

## 9. Conclusions and Perspectives

MERS Coronavirus continues to circulate and infect human species constituting a significant threat with a fatality rate higher than SARS-COV-2. Herein, we summarized the current scientific knowledge about MERS-CoV covering virological, epidemiological, and therapeutic intervention aspects. To our knowledge, MERS-CoV is well-maintained in dromedary camels, resulting in asymptomatic to benign illness. Dromedary camels serve as a major reservoir of the virus with silent spillover human infections. Although dromedary camels are well-known to be the main reservoir of the virus, the origin of the virus and how it was introduced into camels, whether direct or indirect transmission from bats, is not very well understood. Additionally, in the KSA, which comprises 84% of the globally reported cases and 91% of the overall global fatalities, up to 50% of MERS-CoV cases occurred via human-to-human transmission through close contact with asymptomatic or symptomatic individuals infected with MERS-CoV in household or health-care settings. So far, there are no specific drugs or vaccines against MERS disease. Therefore, intensive screening for novel antiviral agents and effective vaccines against MERS-CoV is clearly an urgent need. Finally, MERS is a prime example of a zoonotic disease, and this emphasizes the relevance of a "one health" approach, where efforts of experts in animal health, human health, research, ecology, and epidemiology should come together to control MERS-CoV infections.

## Figures and Tables

**Table 1:** Serological evidence of MERS-CoV prevalence in dromedary and domestic animals in the field.

Study Country	Sampling Year(s)	Sample Size	Assay Applied	Host	Sero-Positivity (%)	Age	Ref.
Australia	2013–14	25	PNA	Dromedary camel	0	Ad/Ju	[26]
Bangladesh	2015	55	PNA	Dromedary camel	31	Ad/Ju	[59]
Bangladesh	2015	18	PNA, ELISA	Sheep	0	Ad	[59]
Chile	2012–13	2	PM, NA	Bactrian camel	0	UK	[46]
Chile	2012–13	25	PM, NA	Alpaca, llama	0	UK	[46]
Netherlands	2012–13	2	PM, NA	Bactrian camel	0	UK	[46]
Netherlands	2012–13	8	PM, NA	Alpaca, llama	0	UK	[46]
Spain “Canary Islands”	2012–13	195	PM, NA	Dromedary camel	14	Ad/Ju	[46]
Spain “Canary Islands”	2015	170	ELISA	Dromedary camel	4.1	UK	[60]
Egypt	2013	110	PNA	Dromedary camel	94	Ad	[29]
Egypt	2013	52	PNA	Dromedary camel	92.3	Ad	[13]
Egypt	1997	43	ELISA, NA	Dromedary camel	81.4	UK	[48]
Egypt	2015–16	1031	NA	Dromedary camel	84.5	Ad/Ju	[58]
Egypt	2014–16	2541	NA	Dromedary camel	71	Ad/Ju	[45]
Egypt	2015–16	145	NA	Sheep, donkey, goat, cattle, buffalo	0.7	Ad	[58]
Egypt	2015–17	409	NA, PNA	Cattle, sheep, goat, donkey, buffalo, and horse	1.7	Ad/Ju	[43]
Egypt	2016–18	2033	NA	Dromedary camel	68.9	Ad/Ju	[54]
Ethiopia	2010–11	188	PM	Dromedary camel	96	Ad	[23]
Iraq	2017	32	NA	Dromedary camel	43.7	Ad/Ju	[54]
Israeli	2013	71	NA, IFA	Dromedary camel	71.8	Ad/Ju	[31]
Israeli	2012–17	411	ELISA, NA	Dromedary camel	62	Ad/Ju	[61]
Israeli	2016	121	ELISA, NA	Alpaca, llama	23.5	UK	[61]
Jordan	2013	11	PM, NA	Dromedary camel	100	Ju	[30]
Jordan	2015–16	304	NA	Dromedary camel	81	Ad/Ju	[54]
Kenya	1993–2013	774	ELISA	Dromedary camel	27.5	UK	[21]
Kenya	2016–2017	1421	ELISA	Dromedary camel	63.7	Ad/Ju	[32]
Mali	2009–10	262	NA, ELISA	Dromedary camel	90	Ad/Ju	[62]
Mongolia	2015	200	PNA	Bactrian camel	0	Ad/Ju	[57]
Nigeria	2010–11	358	PM	Dromedary camel	94	Ad	[23]
Oman	2013	50	PM, NA	Dromedary camel	100	Ad	[46]
Qatar	2013	14	NA	Dromedary camel	100	UK	[50]
KSA	2010–13	310	PNA	Dromedary camel	90	Ad/Ju	[22]
KSA	2014	9	IFA	Dromedary camel	22.2	Ad/Ju	[63]
KSA	2016	171	ELISA	Dromedary camel	84.21	Ad/Ju	[64]
KSA	2017	222	NA	Dromedary camel	81.3	Ad/Ju	[54]
Senegal	2017	72	NA, PNA	Sheep and goat	51.3	Ad/Ju	[43]
Senegal	2017	198	NA	Dromedary camel	65.1	Ad/Ju	[54]
Somalia	1983, 1984	25, 61	ELISA, NA	Dromedary camel	80, 85.2	Ad	[48]
Sudan	1983	60	ELISA, NA	Dromedary camel	86.7	Ad/Ju	[48]
Tunisia	2010–11	204	PM	Dromedary camel	48.5	Ad/Ju	[23]
Tunisia	2016–17	342	NA, PNA	Cattle, sheep, goats, donkey, mule, and horse	1.7	Ad/Ju	[43]
Tunisia	2015–18	782	NA	Dromedary camel	87.3	Ad/Ju	[54]
UAE	2005	151, 500	SPM	Dromedary camel	81.8	Ad	[25]
Uganda	2017	500	NA	Dromedary camel	61.6	Ad/Ju	[54]
USA/Canada	2000–01	6	NA	Dromedary camel	0	Ad	[65]
Ethiopia	2015	632	NA, PNA	Dromedary camel	93.8	Ad/Ju	[66]
Burkina Faso	2015	525	NA, PNA	Dromedary camel	77.1	Ad/Ju	[66]
Morocco	2015	343	NA, PNA	Dromedary camel	74.2	Ad/Ju	[66]
Pakistan	2015–2018	1050	ELISA	Dromedary camel	75	Ad	[67]
Pakistan	2012–2015	565	ELISA	Dromedary camel	39.5	Ad/Ju	[68]
Qatar	2015	15	DPM, PNA	Dromedary camel	100	Ad/Ju	[69]
Qatar	2015	10	DPM, PNA	Alpaca	90	UK	[69]

Abbreviations: PNA (Pseudoparticle Neutralization Assay); NA (Neutralization Assay); IFA (Immunofluorescence Assay); PM (Protein Microarray); SPM (Spike Protein Microarray); ELISA (Enzyme-Linked Immunosorbent Assay); Ad (Adult); Ju (Juvenile); Ad/Ju (Adult and Juvenile); UK (Unknown).
